# Dissection of a *Ciona* regulatory element reveals complexity of cross-species enhancer activity

**DOI:** 10.1016/j.ydbio.2014.03.013

**Published:** 2014-06-15

**Authors:** Wei-Chung Chen, Stefan Pauls, Jamil Bacha, Greg Elgar, Matthew Loose, Sebastian M. Shimeld

**Affiliations:** a Department of Zoology, University of Oxford, South Parks Road, Oxford OX1 3PS, UK; b MRC National Institute for Medical Research, The Ridgeway, Mill Hill, London NW7 1AA, UK; c Centre for Genetics and Genomics, School of Biology, University of Nottingham, Queens Medical Centre, Nottingham NG7 2UH, UK

**Keywords:** *Ciona*, Lens, Crystallin, Evolution, Gene regulatory network

## Abstract

Vertebrate genomes share numerous conserved non-coding elements, many of which function as enhancer elements and are hypothesised to be under evolutionary constraint due to a need to be bound by combinations of sequence-specific transcription factors. In contrast, few such conserved elements can be detected between vertebrates and their closest invertebrate relatives. Despite this lack of sequence identity, cross-species transgenesis has identified some cases where non-coding DNA from invertebrates drives reporter gene expression in transgenic vertebrates in patterns reminiscent of the expression of vertebrate orthologues. Such instances are presumed to reflect the presence of conserved suites of binding sites in the regulatory regions of invertebrate and vertebrate orthologues, such that both regulatory elements can correctly interpret the trans-activating environment. Shuffling of binding sites has been suggested to lie behind loss of sequence conservation; however this has not been experimentally tested. Here we examine the underlying basis of enhancer activity for the *Ciona intestinalis βγ-crystallin* gene, which drives expression in the lens of transgenic vertebrates despite the *Ciona* lineage predating the evolution of the lens. We construct an interactive gene regulatory network (GRN) for vertebrate lens development, allowing network interactions to be robustly catalogued and conserved network components and features to be identified. We show that a small number of binding motifs are necessary for *Ciona βγ-crystallin* expression, and narrow down the likely factors that bind to these motifs. Several of these overlap with the conserved core of the vertebrate lens GRN, implicating these sites in cross species function. However when we test these motifs in a transgenic vertebrate they prove to be dispensable for reporter expression in the lens. These results show that current models depicting cross species enhancer function as dependent on conserved binding sites can be overly simplistic, with sound evolutionary inference requiring detailed dissection of underlying mechanisms.

## Highlights

• Analysis of binding motifs in a *Ciona* enhancer that also works in vertebrate lens.
• Establishment of candidate transcription factors that may regulate this enhancer.
• Construction of a curated, interactive gene regulatory network of lens development.
• Public accessibility of this via a dedicated web site.
• Experimental test of binding motif function in cross species transgenesis.

## Introduction

Whether a gene is expressed or silenced in a particular animal cell depends in part upon interaction between transcription factors expressed by the cell and regulatory DNA associated with the gene. These interactions rely upon the ability of transcription factors to bind to specific DNA sequences, with such binding sites typically focused into stretches of DNA known as enhancers, regulatory elements or Cis-Regulatory Modules (CRMs), amongst other terms. For some genes in some lineages, clustering of binding sites may provide constraint upon sequence evolution, leading to the presence of Conserved Non-coding Elements (CNEs). These can be identified as stretches of DNA associated with orthologous genes that are not part of the canonical transcribed portions of a gene, but are under evolutionary constraint (Woolfe et al., 2005).

Conversely, it is also apparent that in some instances the function of cis-regulatory DNA can be conserved between species without CNEs being easily detectable by sequence comparison. Well-studied examples of this include the CRMs regulating the expression of the *Drosophila even skipped* (*eve*) gene (Ludwig and Kreitman, 1995), and the vertebrate *RET* gene (Fisher et al., 2006a). In these instances, cross-species transgenesis has demonstrated that CRMs from orthologous genes can part-replicate endogenous orthologue expression despite absence of primary sequence conservation. One explanation for such data is maintenance of similar repertoires of transcription factor binding sites in both lineages, such that they preserve the ability to recognise the equivalent trans-activating environment, but that redundancy between sites has allowed their ordering to change. As most binding sites are rather short sequences, primary sequence conservation might hence no longer be detectable. This model of CRM evolution has, however, been tested in only a small number of cases.

A large majority of studies of CNEs have been conducted by comparison between relatively closely related groups of organisms. Jawed vertebrates, for example, share thousands of CNEs (McEwen et al., 2009). However extending such comparisons more broadly across phylogeny has shown that very few vertebrate CNEs are identifiable in invertebrates. For example only a small number have been found conserved with amphioxus (Putnam et al., 2008), a distantly-related chordate, and only isolated cases are identified as conserved between vertebrates and other Phyla (Clarke et al., 2012; Royo et al., 2011). No definitive CNEs have been described as shared between vertebrates and tunicates, the closest living invertebrate relatives to the vertebrates, or between amphioxus and tunicates. The most promising candidates, the CNEs described by Sanges and colleagues, are *Ciona* sequences that show some similarity to vertebrate CNEs (Sanges et al., 2013). However they do not lie in syntenic positions and hence it is debatable as to whether they are homologous and can justifiably be described as conserved. Despite this lack of evidence for definitive CNEs shared between tunicates and other chordates, some evidence for functional similarity of CRMs from orthologous genes in tunicates and amphioxus or vertebrates has been obtained. For example surveys of CNEs identified by sequence comparison between the tunicates *Ciona intestinalis* and *Ciona savignyi* show that some are able to drive reporter expression in vertebrate embryos (Doglio et al., 2013). Studies on specific genes include demonstration of the ability of cis regulatory DNA associated with amphioxus *Hox2* to drive expression in a transgenic tunicate (Wada et al., 2005), and of DNA associated with *C. intestinalis βγ-crystallin* (*Ci-βγcry*) gene to drive expression patterns in vertebrate embryos that resemble the expression of their endogenous orthologues (Shimeld et al., 2005). One interpretation of these studies has been that while primary sequence conservation is not detectable, the CRMs have maintained the ability to interpret the correct cis-regulatory environment via preservation of similar repertoires of binding sites. This hypothesis is yet to be experimentally tested.

Here, we set out to determine if such shared binding sites do explain cross-species CRM function. We focused on the *Ci-βγcry* gene, whose CRM we have previously shown to drive accurate reporter expression in *Ciona* larvae, and which also drives expression in the vertebrate lens where vertebrate βγ-crystallins are expressed. Using serial deletion followed by targeted mutagenesis of specific binding motifs, we show the necessity of several such motifs for correct CRM function in *Ciona*. To understand the vertebrate lens transcriptional environment, we constructed gene regulatory network (GRN) models for the lenses of four vertebrate species. These highlight central roles for several vertebrate genes that could bind to these motifs, and whose *Ciona* orthologues appear as likely candidates for *Ci-βγcry* regulation in *Ciona* larvae. All these data support a role for conserved binding sites in cross species regulation. However when we test the necessity of these motifs via cross-species transgenesis in a vertebrate, none are required for reporter expression in the lens. We conclude that models depicting cross-species function mediated by shared binding sites in the absence of direct experimental verification are overly simplistic, and that understanding such phenomena requires detailed dissection of regulatory elements.

## Materials and methods

### Constructs and *Ciona* transgenesis

Constructs for *Ciona* transgenesis were based on the 1.2 kb upstream sequence of *Ci-βγcry* attached to eGFP as described (Shimeld et al., 2005). Deletion mutants were generated by PCR, and examined via sequencing to confirm deletions and ensure that additional mutations had not been introduced. Binding site mutations were introduced by PCR using modified oligonucleotides, and were similarly verified by sequencing prior to experimental test. *Ciona* transgenics were generated by electroporation of fertilised eggs, essentially as described (Corbo et al., 1997), but using a BTX 830 electroporator. All constructs were tested at least twice in separate experiments, and both positive and negative controls were included to evaluate the success of electroporation and control for background fluorescence (which was never observed). Electroporated embryos were allowed to develop up until the early larval stage. Fluorescent embryos were scored on a Nikon SMZ1500 stereomicroscope fitted with a 2× objective and epifluorescence. In some instances larvae were also examined on a Zeiss Axioskop compound microscope, also equipped with epifluorescence.

### *Ciona* transcription factor gene expression

We used both published data, extracted from the ANISEED database (Tassy et al., 2010), and our analysis of the *Ci-βγcry* regulatory region, to compile a list of candidate transcription factors for *Ci-βγcry* regulation. Expression profiles for these genes were extracted from ANISEED and from published accounts of gene expression. A full account including sources of data is in Supplementary file 1.

### Prediction of transcription factor binding sites

Sequences corresponding to the *Ci-βγcry* gene and adjacent sequence were identified from the *C. intestinalis* and *C. savignyi* genomes (Ensemble location reftig_491:22998–23410) and aligned by Clustalw2 (Larkin et al., 2007). Candidate transcription factor binding sites were identified using MatScan (Blanco et al., 2006) with an initially low threshold of 70% due to the requirement to use vertebrate position weight matrices derived from JASPAR and TRANSFAC (Bryne et al., 2008; Matys et al., 2003). Conservation of sites was considered within the aligned promoter sequences such that at least one of the candidate sites was supported with a threshold of >85%.

### Construction of a vertebrate lens GRN

We used the myGRN database system to construct GRNs for lens development in *Mus musculus*, *Xenopus laevis*, *Gallus gallus* and *Danio rerio* (Bacha et al., 2009). myGRN was built to allow the rapid construction and subsequent interrogation of gene regulatory networks describing developmental processes. Broadly myGRN encompasses the principles first laid out by Davidson et al. (2002) for the identification of gene regulatory interactions. We took a dual approach to identifying interactions from the published literature. Initially, we searched PubMed manually for papers relating to lens development, and entered any interactions described in these papers into myGRN. In a second phase, we identified orthologues for all the genes in the nascent mouse network and entered them as candidate genes in the other three species. We then used myGRN to submit these genes to IHoP and Chilibot, web-accessible services that use natural language processing algorithms to scan paper abstracts and identify putative interactions directly from the text (Chen and Sharp, 2004; Hoffmann and Valencia, 2004). myGRN is able to retrieve results from IHoP and Chilibot searches and make them available for curation. All IHoP results were manually reviewed, and any interactions relevant to lens development were entered into their appropriate networks. We then submitted the interactions retrieved from the literature to Chilibot. Unlike IHoP, Chilibot searches for interactions between a pair of submitted gene names. While IHoP is useful for identifying novel interaction partners for a given gene from the literature, Chilibot is more useful for finding evidence for a given interaction in another species, or finding further evidence for a known interaction. Again, the Chilbot results were manually reviewed using myGRN׳s curation tools, and any interactions relevant to lens were entered. Networks are visualised dynamically from myGRN either using myGRN׳s own tools, or by exporting to YED.

### Zebrafish constructs and transgenics

To generate transgenic zebrafish we used an approach based on the tol2 system (Kawakami et al., 2004). We first amplified the wildtype (WT) and mutated *C. intestinalis* sequences and cloned the PCR products into the pCR8/GW/TOPO vector (Invitrogen). These clones were used as entry clones for inserting the sequences into a tol2 GFP expression vector (Fisher et al., 2006b). Injected zebrafish embryos were raised to maturity and then crossed with WT fish to identify germ line insertions. Lens expression was confirmed in offspring obtained from at least three different founders except for the construct carrying a mutation in the Fox consensus site, for which we identified only two founders.

## Results

### Minimal regulatory region necessary for *Ci-βγcry* expression lies within 315 bp of the transcription start site

**Fig. 1:**
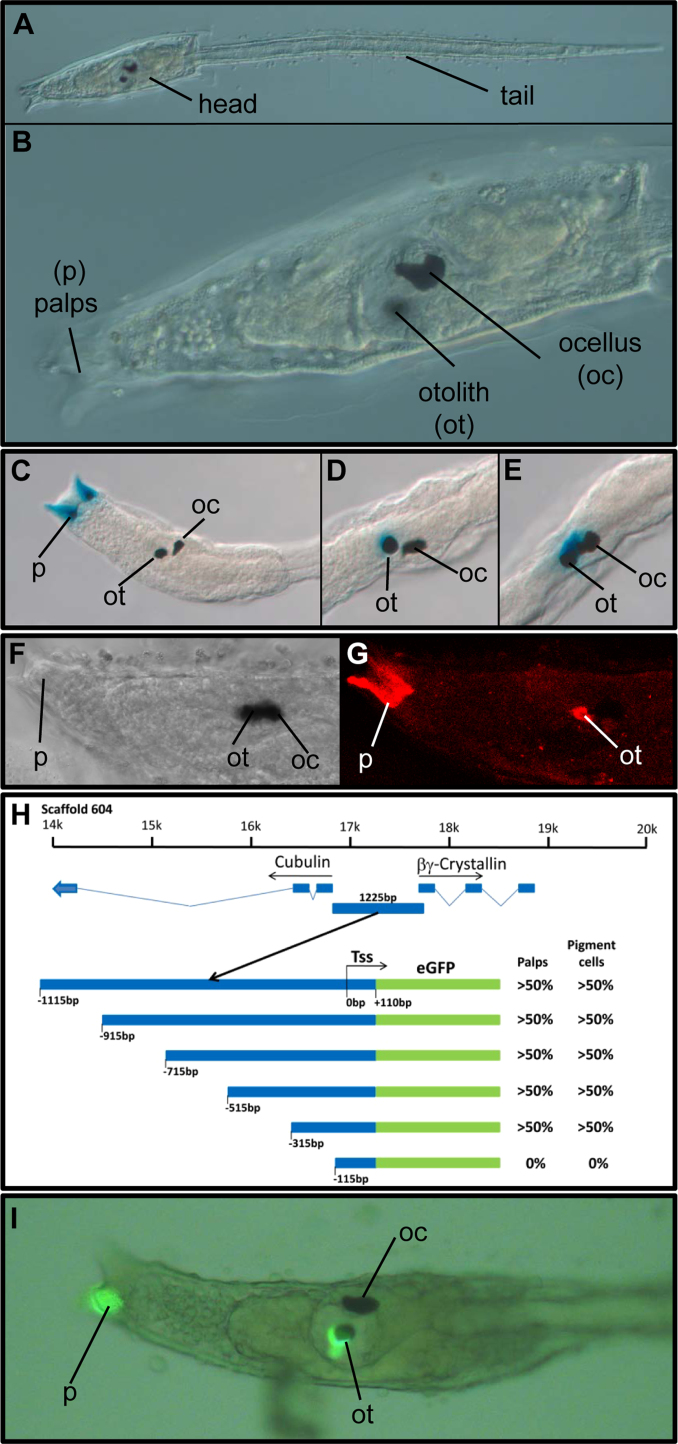
(A) *Ciona* tadpole larva, with anterior to the left. The distinction between head (sometimes described as the trunk) and tail is shown. (B) A magnified view of the head, including the sensory vesicle in which the ocellus (oc) and otolith (ot) pigment cells lie, and the palps (p) at the anterior. (C)–(E**)***Ciona* larvae electroporated with *Ci-βγcrys* pCES. A shows reporter expression in the palps. B shows reporter expression in the otolith. In C, expression includes the otolith but extends more towards the ocellus as described previously (Shimeld et al., 2005). (F, G) Localisation of Ci-βγcrys protein detected by immunohistochemistry. D shows a larval head imaged by DIC microscopy, and E a fluorescent image of the same larva with protein localised in palps and otolith. (H) Initial deletion analysis of the 1225 bp *Ci-βγcrys* 5′ regulatory region. At the top is the location of this region on scaffold 604 of the version 1*C. intestinalis* genome assembly (Dehal et al., 2002), between the divergently transcribed *cubulin* and *Ci-βγcrys* genes. Schematics of successive 200 bp deletions are underneath. Constructs showed transgene expression in palps and pigment cells in at least 50% of embryos (>50%), or no transgene expression was detected in these tissues in any embryo (0%). Each construct was tested in at least 2 independent electroporations, including concurrent positive and negative controls, with positive control transgenesis levels of at least 50% and with at least thirty surviving embryos per construct. TSS indicates the transcription start site, and numbering is from this point. (I) A transgenic larva with fluorescent reporter detected in palp and otolith. Only one palp is labelled, a common occurrence reflecting mosaicism.

The *Ciona* tadpole larva consists of a head (also known as the trunk) which contains the brain, and tail with dorsal neural tube, notochord and axial muscle cells (Fig. 1A and B). Sensory structures in the head include the sensory vesicle, which houses otolith and ocellus pigment cells, and the palps, which also contain secretory cells and which function in settlement site choice and adhesion during metamorphosis (Fig. 1B). Previous study of the *Ci-βγcry* regulatory region was based on a fusion construct in which eGFP was fused in frame with the *Ci-βγcry* start codon, and which was able to drive reporter expression into the palps and pigment cells of *Ciona* larvae (Shimeld et al., 2005). To confirm that this regulatory region could operate independently as a classical enhancer, we cloned it upstream of a basal promoter from a different gene, driving *β-galactosidase* in the vector pCES (Harafuji et al., 2002), and electroporated this construct into *Ciona* zygotes. Reporter expression (Fig. 1C–E) faithfully reproduced endogenous protein localisation (Fig. 1F and G) and original transgene expression (Shimeld et al., 2005).

The original eGFP construct (Shimeld et al., 2005) includes 1225 bp of *Ciona* sequence 5′ to the point of fusion with eGFP. Of this, 1115 bp lies at 5′ to the Transcription Start Site (TSS). Hereafter we refer to the TSS as 0 bp, with sequence 5′ to this denoted with a minus sign (Fig. 1H). To define regulatory sequence within this −1115 bp region, we deleted successive sections of approximately 200 bp from the 5′ end of the construct (Fig. 1H) and tested these via electroporation into *Ciona* zygotes. Deletion of up to 800 bp of 5′ sequence did not affect expression in either the palps or pigment cells (Fig. 1I). However deletion of an additional 200 bp beyond this abolished reporter expression in both sites. These results show that regulatory information necessary to drive reporter expression to both palps and otolith lies within 315 bp of the TSS.

### Sox, Fox, homeodomain and bZip binding motifs are required for *Ci-βγcry* regulation

**Fig. 2:**
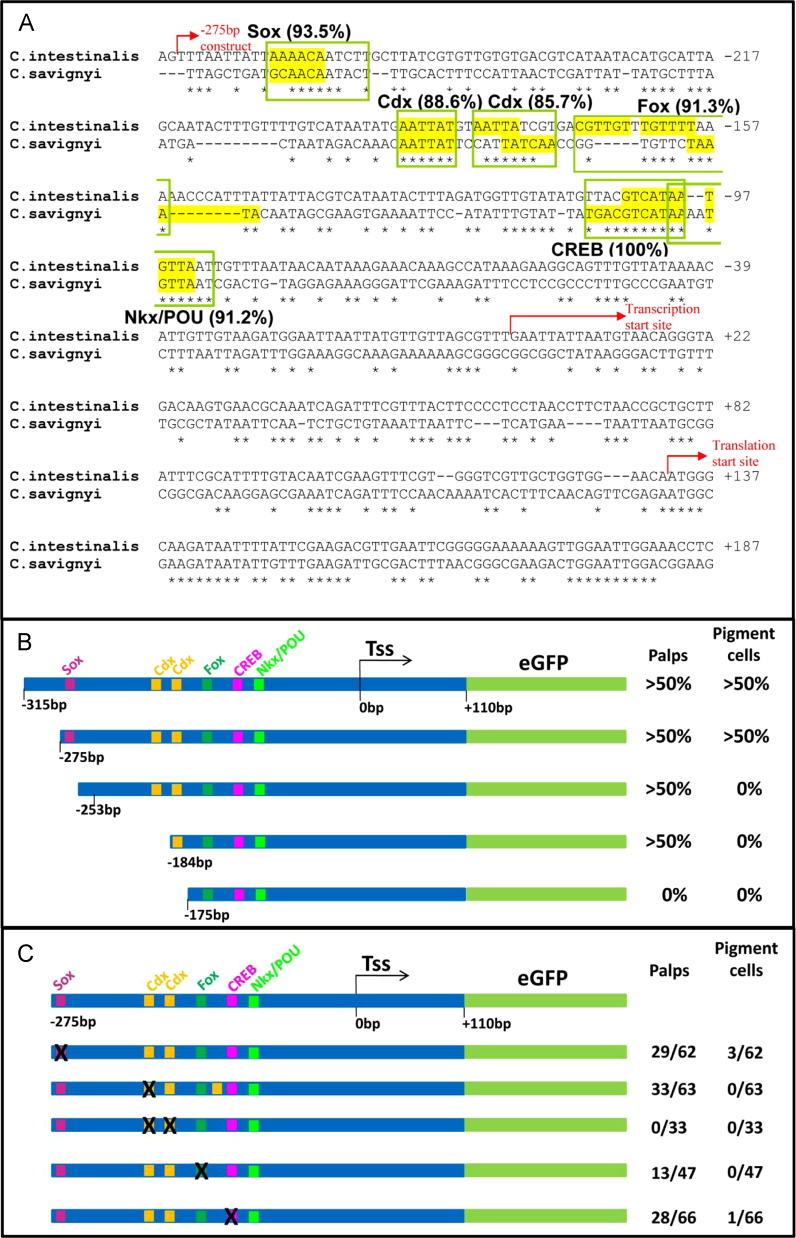
(A) Alignment of the orthologous *Ci-βγcrys* regulatory region from *C. intestinalis* and *C. savignyi*, with putative conserved binding motifs boxed. The names of these reflect the database entry (JASPAR or TRANSFAC) to which they match and do not necessarily mean that the specific factor binds this site in *Ciona*. Highest scores for each site are shown with all sites scoring >70%. Core sequences are highlighted in yellow. The translation start site is shown, as is the presumed transcription start site inferred from the 5′ extreme of cDNA sequence. Numbering starts from this point and refers to the *C. intestinalis* sequence. The start of the minimal −275 bp construct sequence is shown. (B, C) Fine scale deletion and mutation analysis of the *Ciona βγ-crystallin* minimal region defined as in Fig. 1. Presumptive binding motifs are represented as coloured boxes. (B) Successive approximately 20 bp deletions, each tested by electroporation. Only constructs spanning changes in reporter activity are shown. Controls and use of percentage signs are as in Fig. 1. (C) Result of mutagenesis of selected motifs (indicated by a cross). We targeted four motifs, chosen due to potential overlap with the vertebrate lens gene regulatory network (Sox, Fox and CREB) and/or because the deletion experiments indicated a function for the region in which they lay (Sox and Cdx). Results were more variable than for deletions; hence we show the number of reporter-expressing larvae alongside the total number of larvae that developed. Typical transgenesis rates in positive controls were around 50%.

To examine the regulatory landscape of this region more closely, we took two approaches. First we built additional deletion constructs, removing successive regions of approximately 20 bp from the 5′ end of the −315 bp construct. Deletion from −315 bp up to −275 bp did not affect reporter expression, while deletion up until −253 bp abolished palp but not pigment cell expression (Fig. 2B). Deletion of additional approximately 20 bp regions up to −184 bp did not further affect expression, until deletion to −175 bp which abolished all reporter expression (Fig. 2B).

Second we exploited the genome sequence of *C. savignyi* (Small et al., 2007), which is sufficiently distant from *C. intestinalis* to allow constrained non-coding sequences to be identified by sequence comparison. We identified the orthologous locus to *Ci-βγcry* in the *C. savignyi* genome, aligned the equivalent 5′ sequences from both species, and used this to identify fully- or partially-conserved sequences with similarity to known transcription factor binding motifs. This identified motifs for Sox, Fox, CREB and homeodomain proteins (Fig. 2A, B). Comparison of these data with the 20 bp deletion constructs shows that the region containing the Sox binding motif is required for pigment cell expression, and the region containing the second of paired Cdx motifs (a short motif better considered as a more general homeodomain motif) is required for palp expression, while the region including the Sox binding motif is dispensable for palp expression (Fig. 2B, C).

To more precisely examine the role of individual predicted binding motifs, we separately mutated selected motifs in the context of the −275 bp construct (Fig. 2C). Mutation of the Sox motif did not affect palp expression, but reduced pigment cell expression to a low level (Fig. 2C). Mutation of the first Cdx motif also left palp expression unaffected, but abolished pigment cell expression, whereas mutation of both Cdx motifs abolished all reporter expression. Mutation of the Fox motif reduced palp expression and abolished pigment cell expression, while mutation of the CREB motif did not affect palp expression but reduced pigment cell expression to a very low level (Fig. 2C). These data show that Sox, Fox, CREB and Cdx motifs are all required for normal reporter expression, implying they act in vivo to regulate *Ci-βγcry* expression via binding of Sox, Fox, bZip and homeodomain factors.

### Refining hypotheses of *Ci-βγcry* regulation with gene expression data

Since mutation of binding sites reduced expression rather than yielding ectopic expression, regulators binding to them are likely to be activators rather than repressors. We hence reasoned that to be responsible for activation in vivo, the genes encoding such regulators are likely to be co-expressed with *Ci-βγcry*. The ANISSED database (Tassy et al., 2010) maintains a carefully evaluated atlas of *Ciona* gene expression, mapped onto ontogeny and including the results of an extensive survey of transcription factor gene expression (Satou et al., 2002). We first searched this database for transcription factor genes expressed in the same territories as *Ci-βγcry*, the otolith pigment cell and palps. *Ciona* has two pigment cells in the sensory vesicle, the ocellus and the otolith, while the palps are anterior ectodermal protrusions containing sensory neurons and secretory cells. Since the detection of standard in situ stain can be challenging in the pigmented cells, possibly leading to under-reporting of pigment cell expression, we also expanded the search to consider the whole sensory vesicle. We supplemented these searches with consideration of other published expression patterns not in the ANISEED database, and further tested some genes by in situ hybridisation. We considered both tadpole larvae, when *Ci-βγcry* is expressed in both palps and otolith (Shimeld et al., 2005), and late tail bud embryos, when expression *Ci-βγcry* initiates in the palps but which are prior to the onset of otolith expression. The results of these searches can be seen in Table 1 and Supplementary file 1.

Seven homeobox genes were identified with evidence of expression in the palps. Only three homeobox genes show good evidence of expression in the pigment cells: *Pax6*, *Rx* and *Six3/6*, all orthologues of genes involved in the visual system in vertebrates (D’Aniello et al., 2006; Hamada et al., 2011; Irvine et al., 2008; Mazet et al., 2005). However 16 homeobox genes were identified with evidence of expression in the sensory vesicle, and it remains possible that several of these will be expressed in the pigment cells. A smaller number of Fox, Sox and bZIP genes were also identified, including FoxB and FoxC, SoxB1 and SoxC, and CREB and xBPa, respectively. Amongst these are genes whose vertebrate orthologues are predicted by our lens GRN (see below) to regulate *βγ-crystallins*.

### Construction of vertebrate lens GRNs

**Fig. 3:**
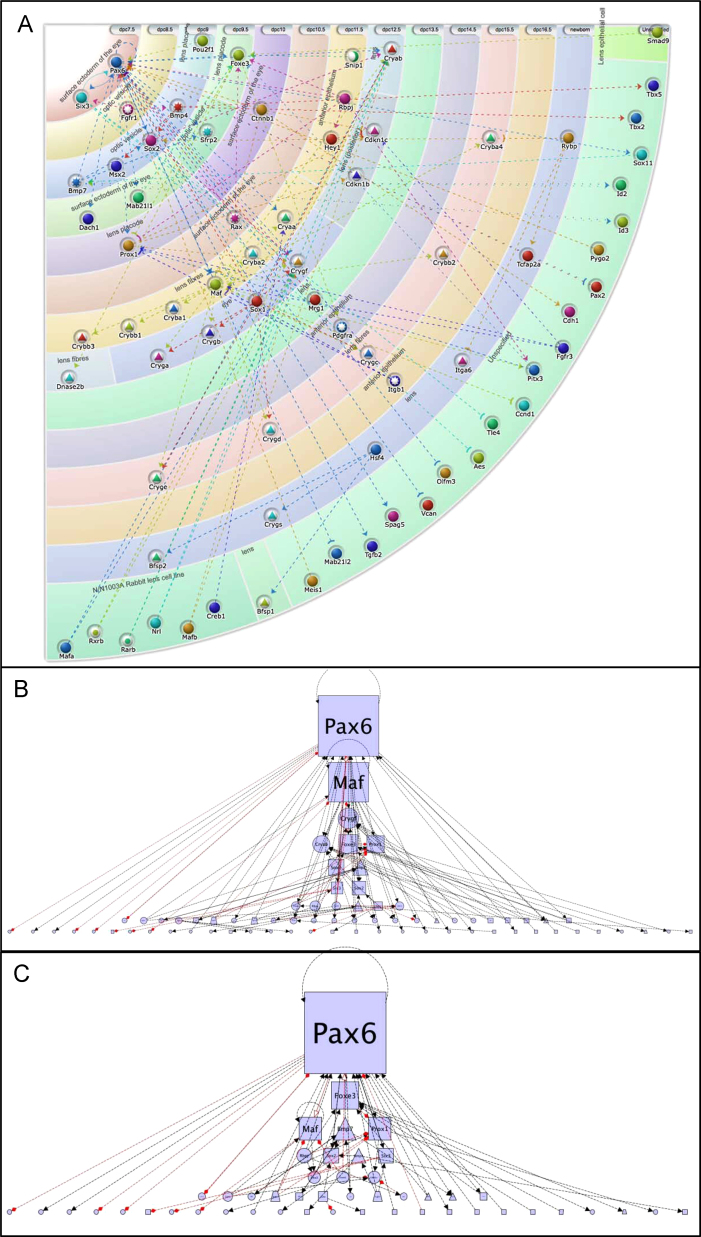
(A) The complete vertebrate mouse lens network as illustrated by myGRN. Developmental time flows from top left (7.5 dpc) to bottom right. The outermost layer of the network includes all those interactions that cannot be placed in a specific tissue or time point. Importantly genes appear only once within the network and so interactions that feed forward or back can be clearly seen. Dashed lines indicate interactions for which the existence of intermediates cannot be formally excluded. The circular symbols represent the type of gene: solid circles are transcription factors, solid asterisks are signals, inverted asterisks represent receptors and triangles illustrate terminal markers. This network is available in a fully interactive format at http://public.networks.mygrn.org/. An animated version of this network is provided as Supplementary movie 1. (B, C) Here the complete network is visualised with genes sized according to centrality within the network. Genes with more connections are larger. When including the crystallins, Pax6 and Maf emerge as key regulators (B). However, if the crystallins are excluded from the network, the relative importance of Maf is decreased and Foxe3 appears more important (C).

It has been previously reported that the full 1255 bp *Ci-βγcry* construct can drive reporter expression in the lens of a transgenic vertebrate (Shimeld et al., 2005). While this expression domain maps well onto the expression of vertebrate *βγ-crystallins*, interpretation is complex since *Ciona* itself lacks a lens and indeed the tunicate lineage is hypothesised to have separated from the lineage leading to vertebrates before the lens evolved (Shimeld and Holland, 2000). Hence, to guide interpretation of cross-species transgenesis, we constructed GRN models of vertebrate lens development, using the myGRN suite of tools (Bacha et al., 2009). Whilst lens development has been studied in a range of model vertebrates, the mouse remains the best-characterised in terms of molecular interactions (Ogino et al., 2012). We therefore first curated a network for the mouse by extensive mining of the published literature. In total, the mouse lens network contains 73 genes with 118 interactions among them (Fig. 3A – view online at http://goo.gl/OAvZyC). Of these 73 genes, 46 were involved in interactions that could be localised to a specific embryological tissue and time of development. The remaining interactions have been defined in cell lines or in vitro assays and so cannot be placed within a specific developmental time point or tissue. As previously reported (Ogino et al., 2012), Pax6 emerges as a crucial regulator within the GRN with 18 targets and 14 upstream activators/repressors. Similarly, Maf transcription factors play an integral role within the network with 16 targets and 4 upstream activators/repressors. These measures of centrality within the network may be elevated as a consequence of the expansion of crystallin family members within vertebrates (Lovicu and Robinson, 2004), or by bias resulting from some transcription factors being more extensively studied than others. The myGRN system can be used to remove elements from the network. Exclusion of the crystallins still places Pax6 as a crucial regulator, but the relative importance of Maf is decreased, with Foxe3 now placed with more prominence (compare Fig. 3B with Fig. 3C).

Supplementary material related to this article can be found online at doi:10.1016/j.ydbio.2014.03.013.

The following is the Supplementary material related to this article Video 1.

Video 1 .

To investigate the evolutionary conservation of the interactions in the mouse network we asked which interactions were supported by data from other model systems. We used the interactions from mouse as seed networks to investigate the frog, chick and fish, utilising the tools available within myGRN to generate networks for each (Bacha et al., 2009). The frog network contained 31 genes with 42 interactions, the fish 12 genes with 8 interactions and the chick 37 genes with 66 interactions. Across these networks, 16 interactions were supported by direct evidence from the literature in more than one species, and key transcriptional regulators emerging from this comparison included Creb1, Foxe3, Meis1, Pax6, Prox1 and Six3 (Supplementary Table 1).

**Fig. 4:**
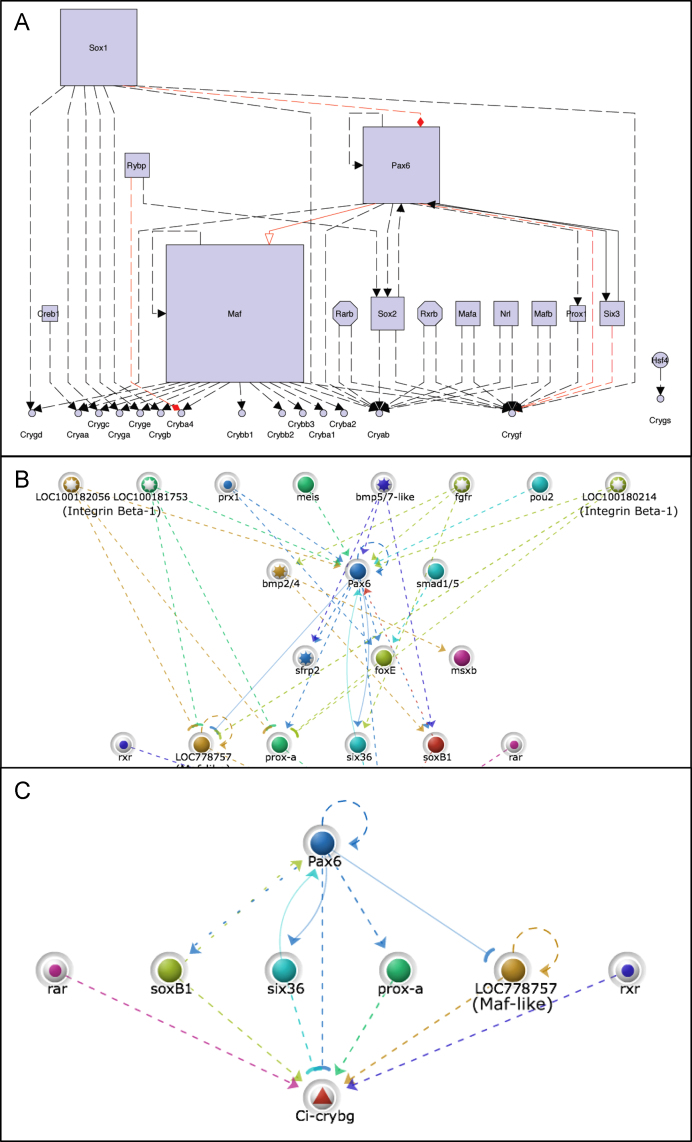
(A) The network now shows only those genes with direct connexions to the crystallins. The size of the symbol representing each gene is proportional to the number of connexions it makes in the network. (B) A hypothetical Ciona ‘lens’ network derived from interactions occurring in the mouse. Note that several different candidates exist in Ciona for Integrin Beta-1 like. (C) The network now highlights those interactions required for the expression of *Ci-βγcry,* as predicted by myGRN based on the mouse lens network.

We next generated the subnetwork identifying those factors upstream of the mouse crystallins (Fig. 4A). Candidate direct regulators of the crystallins included Pax6, Six3, Maf family members, Nrl, RARs, SoxB1, Creb1, Hsf4 and Prox1. This core network describing vertebrate lens development can be used to generate a prediction of possible network topologies in *Ciona* that may regulate the *βγ-crystallins*. The myGRN database allows the transposition of networks from one species to another based on sequence orthology, allowing the generation of testable hypothesis based on known characterised interactions in other species (Supplementary Tables 2 and 3). The complete mouse network transposed to *Ciona* highlights many of the upstream factors previously identified by binding motif analysis and consideration of *Ciona* gene expression profiles (Fig. 4B). By focussing on *Ci-βγcry* alone we obtain a hypothetical network for further analysis (Fig. 4C).

### *Ciona βγ-crystallin* binding motif function assessed in transgenic vertebrates

**Fig. 5:**
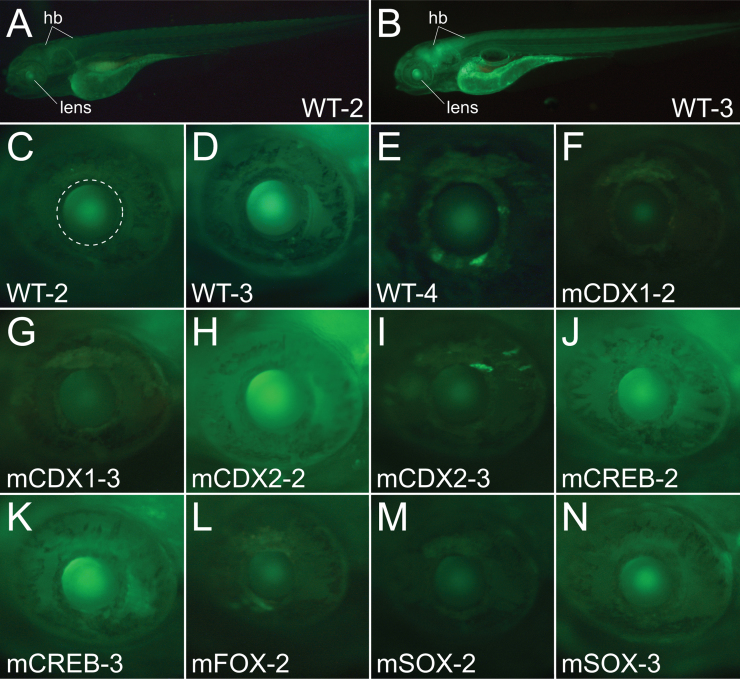
GFP in zebrafish lens driven by the *Ci-βγcry* −275 bp construct (designated WT) and mutant binding site constructs. (A, B) Wild-type (WT) −275 bp constructs in two lines showing the whole fish. The lens is shown, as is the hindbrain (hb). (C, E) Wild-type (WT) −275 bp constructs in three separate lines, showing just the eye with the lens circled in C as a reference. (F)–(N) Lines with constructs with mutant (m) binding motifs as in Fig. 2C; mCDX1 (first Cdx motif mutated, 2 lines). mCDX2 (both Cdx motifs mutated, 2 lines). mCREB (CREB motif mutated, 2 lines). mFOX (Fox motif mutated, 1 line). mSOX (Sox motif mutated, 2 lines).

The GRNs describing vertebrate lens development include a core set of vertebrate transcription factors, many of which directly or indirectly regulate the *βγ-crystallins*, and whose *Ciona* orthologues appear as candidate regulators of *Ci-βγcry* as identified by binding motif analysis and *Ciona* gene expression profiles. These genes are obvious candidates for the cross-species activity of the *Ci-βγcry* regulatory element. To test this, we examined the ability of *Ci-βγcry* constructs carrying mutations in binding motifs to drive reporter expression in transgenic zebrafish. Since high-throughput co-injection strategies in zebrafish produce a high degree of mosaicism and render weak enhancer activity difficult to assess we used Tol2 transgenesis, establishing multiple independent lines for each construct and considering only congruent reporter patterns. We first tested the complete *Ci-βγcry* −275 bp minimal enhancer. This confirmed its ability to drive reporter expression in the lens, and expression was also consistently detected in the CNS (Fig. 5A–E). Lens expression was weak compared to endogenous lens enhancers like those derived from zebrafish *Sox21* (Pauls et al., 2012), and we noted some variation in reporter strength (compare Fig. 5D and E). This shows that the *Ci-βγcry* minimal enhancer is a weak activator in the zebrafish, and prone to positional insertion effects, but drives reporter expression in the lens as previously reported for the full 1225 bp construct in transgenic *Xenopus* (Shimeld et al., 2005).

When, however, we tested if constructs with mutant binding motifs were still capable of driving lens expression in transgenic zebrafish embryos, all were capable of doing so, with expression levels and patterns similar to those of the wild type construct (Fig. 5F–N). This shows that each binding motif, despite being required for correct reporter expression in *Ciona*, contributes at most a weak effect on cross-species expression. We conclude that, whilst the overall ability of the *Ci-βγcry* minimal element to drive reporter expression into appropriate areas of widely divergent embryos appears to be conserved, this is not mediated by any of the single binding motifs essential for reporter expression in *Ciona*.

## Discussion

While vertebrates share many CNEs, very few are shared with any invertebrate and none with sea squirts, members of the closest living invertebrate lineage to the vertebrates. However a few reports demonstrate that some invertebrate CRMs, including some from sea squirts, can drive appropriate reporter gene expression in a transgenic vertebrate (Doglio et al., 2013; Sanges et al., 2013). Such findings have led to considerable discussion as to the nature and evolutionary meaning of such apparent functional conservation in the absence of primary sequence conservation. In this study, we have combined GRN construction with experimental deconstruction of CRM function to dissect the nature of cross species activity of a CRM from the sea squirt *Ciona*.

### Constructing a vertebrate lens GRN: evolutionary and developmental insights

By mining published literature we have constructed a GRN of vertebrate lens development in which each connection can be assessed according to type of validating data that support it. This covers and extends the previously reported GRNs (Ogino et al., 2012), as well as extending the analysis to multiple vertebrate species. This last point is significant, as it allows interactions found in multiple vertebrate species to be identified. These are likely to be conserved, ancestral interactions, distinguishing them from interactions confined to single vertebrate models which are likely to be more recent evolutionary innovations. This network is publicly available at http://public.networks.mygrn.org/ (Bacha et al., 2009) in an interactive form such that users can modulate the network by focusing on specific stages or genes, removing nodes, and viewing underlying supporting data.

Developmental insight comes from the identification of network properties. First is the centrality, as measured by the number of interactions, of a small number of key transcription factors in the network, including Pax6 and Maf. This was also identified in the network constructed by Ogino et al. (2012). One problem with such metrics is acquisition bias, i.e. genes that are more highly studied tend to have more identified connections and hence to appear more important. A case in point here is that the direct regulation of several crystallin genes has been very well studied, and hence the importance of their immediate regulators may tend to be over-emphasised in the network. Using myGRN, we can remove individual genes from the network, allowing this to be assessed. Such manipulations show that Maf may owe its apparent importance to this effect, while Pax6 remains a key regulator whether the crystallins are included or excluded. Foxe3 gains in relative importance when crystallins are removed.

Second, comparison between vertebrates identifies shared properties, that is interactions (and hence genes) operating in a similar way in different vertebrate species. Both *Pax6* and *Foxe3* appear in this gene list, along with several other transcription factor genes. These genes are strong candidates for a conserved, vertebrate-wide lens GRN. Many of these apparently conserved interactions have so far been identified only in subsets of vertebrates. We suggest these are good candidates for further study, as our GRN predicts they are likely to be more widely conserved.

Third we note that a number of regulatory loops appear in the network, including autoregulation by *Pax6* and *Maf*, and positive feedback between *Pax6* and *Six3*. These loops confer robustness on crystallin gene expression, such that once the crystallin regulators have been established, they are maintained in these cells. Mechanisms that lock in expression of genes required for the ultimate fate of a cell are clearly of broad importance particularly when considering redirection of cell fate. Finally the translation of the vertebrate lens network to *Ciona* based on the maintenance of key interactions required for the activation of crystallins does predict regulators of *Ci-βγcry* which can be confirmed in *Ciona* by the presence of candidate binding sites in the promoter sequence.

### Regulation of *Ci-βγcry* in *C. intestinalis*

Deletion analysis, plus identification of conserved binding motifs and their functional validation by mutagenesis, has identified candidate regulators for *Ci-βγcry* gene expression. Sox, Fox, homeodomain and bZip binding motifs are all required for the correct expression of *Ci-βγcry* in the otolith pigment cell of the sensory vesicle, while only the twin homeodomain motifs are required for expression in the palps. Gene expression data exclude many members of these transcription factor classes from a role in regulating *Ci-βγcry* in vivo, as they are not expressed in the same cells. Of the remaining genes we have not directly demonstrated that they regulate *Ci-βγcry*: this would require at least knocking down these genes, something technically feasible in *Ciona* using injection of antisense morpholino oligonucleotides into fertilised eggs, but unviable in this context due to the relatively late stage of expression of *Ci-βγcry* and pleiotropic roles of most transcription factors, manifesting in earlier defects when knocked down and hence masking later developmental events (Imai et al., 2009). Some candidate regulators do make sense in the context of the vertebrate lens GRN, including Pax6, Six3/6 and CREB. If these factors are responsible for regulating the *Ci-βγcry* CRM in both *Ciona* and vertebrate lens cells, then this would support a model in which a conserved, ancestral regulatory network has been inherited by both vertebrate and tunicate lineages, with its co-option into lens construction occurring in vertebrates only, and with CRM sequence identity lost due to shuffling of binding sites. However further investigation suggests that this is too simple an explanation.

### Evaluating models of cross-species conservation of CRM function

Our results show that models depicting the ability of CRMs to function in the absence of primary sequence conservation over wide phylogenetic distances as dependent on conserved binding sites are likely to be overly simplistic, at least in this case. This is despite several lines of evidence appearing to support such a model. To reiterate these: (i) dissection of the *Ci-βγcry* regulatory element in *Ciona* illustrates the necessity for several binding motifs for correct function, while gene expression data point to the likely trans-acting factors involved; (ii) vertebrate lens GRNs highlight some of the same factors as involved in regulating the orthologous vertebrate genes in the lens; and (iii) this is the structure in which the *Ci-βγcry* regulatory element is able to drive expression in transgenic vertebrates. Taken together, these data fit the predictions of a binding site model, suggesting that they reflect conserved regulatory interaction between several trans-acting factors and the *Ci-βγcry* regulatory region, inherited at least in part by both tunicate and vertebrate lineages but with sequence identity lost as binding sites shuffle.

Two factors suggest that this interpretation is too simplistic. First, not all identified similarities in trans-acting factors between the vertebrate lens network and *Ci-βγcry* regulation involve orthologues. Many do (for example *SoxB*1 and some of the homeobox genes) but others do not (for example FoxE in the lens network, but other Fox families are better candidates in *Ciona*). Orthologues provide firmer evidence for conservation. The model also predicts that these binding motifs should function in a cross-species context. When we tested this, none had any impact on transgene expression. This shows that we cannot look just to these motifs as an explanation for cross species enhancer activity. More complex explanations for the ability of the *Ci-βγcry* regulatory region to drive expression in transgenic vertebrates are possible. For example we could hypothesise that no single motif is necessary, but that the sum of their activities is sufficient for transgene expression. This might be revealed by more complex mutagenesis–transgenesis experiments in which multiple binding motifs were simultaneously mutated. Or we could hypothesise that cryptic sites for these or other as yet unidentified factors present in the minimal *Ci-βγcry* regulatory region are responsible for transgene expression, including the possibility that unidentified low affinity sites, insufficient for activation in *Ciona* but sufficient in vertebrates, drive lens expression in mutated constructs. Only this last possibility (the presence of cryptic binding sites for orthologous transcription factors that function in the fish lens but not in *Ciona* due to differences in binding affinities between site and trans-acting factor in the two lineages) would support conservation of mechanism. It seems unlikely that all sites would behave in this way, being necessary in *Ciona* but redundant to cryptic sites in vertebrates. In general then, simple models explaining cross-species enhancer activity operating via the same mechanism in different lineages become unviable. In turn, this means assuming that cross-species enhancer activity reflecting a shared ancestral mechanism is unsafe and consequently extrapolation from such models to the understanding of broader evolutionary situations, such as the evolution of the structures in which such genes are expressed, becomes tenuous. We thus suggest that the evolutionary models based on the inference of shared, regulatory interactions from transgene data where regulatory details have not been established should be treated with caution, and suggest that mechanistic details need to be understood if a firm foundation for evolutionary hypotheses is to be achieved.

## Figures and Tables

**Table 1.** .

**Gene**	**Palp expression**		**Sensory vesicle expression**		**In or adjacent to otolith**
	**Late tail bud**	**Tadpole larva**	**Late tail bud**	**Tadpole larva**	**Tadpole larva**
**Homeobox genes**					
**DllB**	Yes	Yes	No	No	ND
**DllC**	Yes	Yes	No	No	Nd
**Isl1**	No?	Yes	Yes	Yes	Nd
**Lhx1**	No	Nd	Yes	Nd	Nd
**Lhx3/4**	No	No	Yes	Nd	Nd
**Irx-B**	Nd	Yes	Nd	Nd	Nd
**Otx**	Yes	Nd	Yes	Nd	Nd
**Pbx**	Yes	Yes	Yes	Yes	Nd
**MEIS**	No	No	Yes	Yes	Nd
**Arx**	No	No	Yes	Nd	Nd
**Barx-a**	Yes	Nd	No	Nd	Nd
**Gsx**	No	No	Yes	Yes	Nd
**Hox1**	No	No	Yes	No	Nd
**Pax3/7**	No	No	Yes	No	Nd
**Pax6**	No	No	Yes	Yes	Yes
**Prop**	No	Nd	Yes	Nd	Nd
**ProxA**	No	Nd	Yes	Nd	Nd
**Rx**	No	No	Yes	Yes	Yes
**Msx**	No	No	Yes	Yes	Nd
**Six3/6**	No	No	Yes	Yes	Yes

**Fox genes**					
**FoxC**	Yes	Yes	Yes	Nd	Nd
**FoxB**	No	Nd	Yes	Nd	Nd
**FoxP**	Yes	Nd	Yes	Nd	Nd
**FoxHb**	Yes	Yes	Nd	Nd	Nd

**Sox genes**					
**SoxB1**	No	No	Yes	Yes	Yes
**SoxC**	Yes	Nd	Yes	Nd	Nd
**SoxE**	Yes	Nd	Yes	Nd	Nd

**bZIP genes**					
**CREB/ATFa**	Yes	Nd	No	Nd	Nd
**CREB1/ ATF1**	Yes	Nd	No	Nd	Nd
**xBPa**	Nd	ND	Yes	Yes	Yes

## References

[bib1] Bacha J., Brodie J.S., Loose M.W. (2009). myGRN: a database and visualisation system for the storage and analysis of developmental genetic regulatory networks. BMC Dev. Biol..

[bib2] Blanco E., Farre D., Alba M.M., Messeguer X., Guigo R. (2006). ABS: a database of Annotated regulatory Binding Sites from orthologous promoters. Nucleic Acids Res..

[bib3] Bryne J.C., Valen E., Tang M.H., Marstrand T., Winther O., da Piedade I., Krogh A., Lenhard B., Sandelin A. (2008). JASPAR, the open access database of transcription factor-binding profiles: new content and tools in the 2008 update. Nucleic Acids Res..

[bib4] Chen H., Sharp B.M. (2004). Content-rich biological network constructed by mining PubMed abstracts. BMC Bioinform..

[bib5] Clarke S.L., VanderMeer J.E., Wenger A.M., Schaar B.T., Ahituv N., Bejerano G. (2012). Human developmental enhancers conserved between deuterostomes and protostomes. PLoS Genet..

[bib6] Corbo J.C., Levine M., Zeller R.W. (1997). Characterization of a notochotd-specific enhancer from the *Brachyury* promoter region of the ascidian, *Ciona intestinalis*. Development.

[bib7] D’Aniello S., D’Aniello E., Locascio A., Memoli A., Corrado M., Russo M.T., Aniello F., Fucci L., Brown E.R., Branno M. (2006). The ascidian homolog of the vertebrate homeobox gene *Rx* is essential for ocellus development and function. Differentiation.

[bib8] Davidson E.H., Rast J.P., Oliveri P., Ransick A., Calestani C., Yuh C.H., Minokawa T., Amore G., Hinman V., Arenas-Mena C., Otim O., Brown C.T., Livi C.B., Lee P.Y., Revilla R., Schilstra M.J., Clarke P.J., Rust A.G., Pan Z., Arnone M.I., Rowen L., Cameron R.A., McClay D.R., Hood L., Bolouri H. (2002). A provisional regulatory gene network for specification of endomesoderm in the sea urchin embryo. Dev. Biol..

[bib9] Dehal P., Satou Y., Campbell R.K., Chapman J., Degnan B., De Tomaso A., Davidson B., Di Gregorio A., Gelpke M., Goodstein D.M., Harafuji N., Hastings K.E., Ho I., Hotta K., Huang W., Kawashima T., Lemaire P., Martinez D., Meinertzhagen I.A., Necula S., Nonaka M., Putnam N., Rash S., Saiga H., Satake M., Terry A., Yamada L., Wang H.G., Awazu S., Azumi K., Boore J., Branno M., Chin-Bow S., DeSantis R., Doyle S., Francino P., Keys D.N., Haga S., Hayashi H., Hino K., Imai K.S., Inaba K., Kano S., Kobayashi K., Kobayashi M., Lee B.I., Makabe K.W., Manohar C., Matassi G., Medina M., Mochizuki Y., Mount S., Morishita T., Miura S., Nakayama A., Nishizaka S., Nomoto H., Ohta F., Oishi K., Rigoutsos I., Sano M., Sasaki A., Sasakura Y., Shoguchi E., Shin-i T., Spagnuolo A., Stainier D., Suzuki M.M., Tassy O., Takatori N., Tokuoka M., Yagi K., Yoshizaki F., Wada S., Zhang C., Hyatt P.D., Larimer F., Detter C., Doggett N., Glavina T., Hawkins T., Richardson P., Lucas S., Kohara Y., Levine M., Satoh N., Rokhsar D.S. (2002). The draft genome of *Ciona intestinalis*: insights into chordate and vertebrate origins. Science.

[bib10] Doglio L., Goode D.K., Pelleri M.C., Pauls S., Frabetti F., Shimeld S.M., Vavouri T., Elgar G. (2013). Parallel evolution of chordate cis-regulatory code for development. PLoS Genet..

[bib11] Fisher S., Grice E.A., Vinton R.M., Bessling S.L., McCallion A.S. (2006). Conservation of RET regulatory function from human to zebrafish without sequence similarity. Science.

[bib12] Fisher S., Grice E.A., Vinton R.M., Bessling S.L., Urasaki A., Kawakami K., McCallion A.S. (2006). Evaluating the biological relevance of putative enhancers using Tol2 transposon-mediated transgenesis in zebrafish. Nat. Protoc..

[bib13] Hamada M., Shimozono N., Ohta N., Satou Y., Horie T., Kawada T., Satake H., Sasakura Y., Satoh N. (2011). Expression of neuropeptide- and hormone-encoding genes in the *Ciona intestinalis* larval brain. Dev. Biol..

[bib14] Harafuji N., Keys D.N., Levine M. (2002). Genome-wide identification of tissue-specific enhancers in the *Ciona* tadpole. Proc. Natl. Acad. Sci. USA.

[bib15] Hoffmann R., Valencia A. (2004). A gene network for navigating the literature. Nat. Genet..

[bib16] Imai K.S., Stolfi A., Levine M., Satou Y. (2009). Gene regulatory networks underlying the compartmentalization of the *Ciona* central nervous system. Development.

[bib17] Irvine S.Q., Fonseca V.C., Zompa M.A., Antony R. (2008). Cis-regulatory organization of the *Pax*6 gene in the ascidian *Ciona intestinalis*. Dev. Biol..

[bib18] Kawakami K., Takeda H., Kawakami N., Kobayashi M., Matsuda N., Mishina M. (2004). A transposon-mediated gene trap approach identifies developmentally regulated genes in zebrafish. Dev. Cell.

[bib19] Larkin M.A., Blackshields G., Brown N.P., Chenna R., McGettigan P.A., McWilliam H., Valentin F., Wallace I.M., Wilm A., Lopez R., Thompson J.D., Gibson T.J., Higgins D.G. (2007). Clustal W and Clustal X version 2.0. Bioinformatics.

[bib20] Lovicu F.J., Robinson M.L. (2004). Development of the Ocular Lens.

[bib21] Ludwig M.Z., Kreitman M. (1995). Evolutionary dynamics of the enhancer region of even-skipped in *Drosophila*. Mol. Biol. Evol..

[bib22] Matys V., Fricke E., Geffers R., Gossling E., Haubrock M., Hehl R., Hornischer K., Karas D., Kel A.E., Kel-Margoulis O.V., Kloos D.U., Land S., Lewicki-Potapov B., Michael H., Munch R., Reuter I., Rotert S., Saxel H., Scheer M., Thiele S., Wingender E. (2003). TRANSFAC: transcriptional regulation, from patterns to profiles. Nucleic Acids Res..

[bib23] Mazet F., Hutt J.A., Millard J., Graham A., Shimeld S.M. (2005). Molecular evidence from *Ciona intestinalis* for the evolutionary origin of vertebrate cranial placodes. Dev. Biol..

[bib24] McEwen G.K., Goode D.K., Parker H.J., Woolfe A., Callaway H., Elgar G. (2009). Early evolution of conserved regulatory sequences associated with development in vertebrates. PLoS Genet..

[bib25] Ogino H., Ochi H., Reza H.M., Yasuda K. (2012). Transcription factors involved in lens development from the preplacodal ectoderm. Dev. Biol..

[bib26] Pauls S., Smith S.F., Elgar G. (2012). Lens development depends on a pair of highly conserved *Sox*21 regulatory elements. Dev. Biol..

[bib27] Putnam N.H., Butts T., Ferrier D.E.K., Furlong R.F., Hellsten U., Kawashima T., Robinson-Rechavi M., Shoguchi E., Terry A., Yu J.K., Benito-Gutierrez E., Dubchak I., Garcia-Fernandez J., Gibson-Brown J.J., Grigoriev I.V., Horton A.C., de Jong P.J., Jurka J., Kapitonov V.V., Kohara Y., Kuroki Y., Lindquist E., Lucas S., Osoegawa K., Pennacchio L.A., Salamov A.A., Satou Y., Sauka-Spengler T., Schmutz J., Shin-I T., Toyoda A., Bronner-Fraser M., Fujiyama A., Holland L.Z., Holland P.W.H., Satoh N., Rokhsar D.S. (2008). The amphioxus genome and the evolution of the chordate karyotype. Nature.

[bib28] Royo J.L., Maeso I., Irimia M., Gao F., Peter I.S., Lopes C.S., D’Aniello S., Casares F., Davidson E.H., Garcia-Fernandez J., Gomez-Skarmeta J.L. (2011). Transphyletic conservation of developmental regulatory state in animal evolution. Proc. Natl. Acad. Sci. USA.

[bib29] Sanges R., Hadzhiev Y., Gueroult-Bellone M., Roure A., Ferg M., Meola N., Amore G., Basu S., Brown E.R., De Simone M., Petrera F., Licastro D., Strahle U., Banfi S., Lemaire P., Birney E., Muller F., Stupka E. (2013). Highly conserved elements discovered in vertebrates are present in non-syntenic loci of tunicates, act as enhancers and can be transcribed during development. Nucleic Acids Res..

[bib30] Satou Y., Takatori N., Fujiwara S., Nishikata T., Saiga H., Kusakabe T., Shin-i T., Kohara Y., Satoh N. (2002). Ciona intestinalis cDNA projects: expressed sequence tag analyses and gene expression profiles during embryogenesis. Gene.

[bib31] Shimeld S.M., Holland P.W.H. (2000). Vertebrate innovations. Proc. Natl. Acad. Sci. USA.

[bib32] Shimeld S.M., Purkiss A.G., Dirks R.P.H., Bateman O.A., Slingsby C., Lubsen N.H. (2005). Urochordate ßG-crystallin and the evolutionary origin of the vertebrate eye lens. Curr. Biol..

[bib33] Small K.S., Brudno M., Hill M.M., Sidow A. (2007). A haplome alignment and reference sequence of the highly polymorphic *Ciona savignyi* genome. Genome Biol..

[bib34] Tassy O., Dauga D., Daian F., Sobral D., Robin F., Khoueiry P., Salgado D., Fox V., Caillol D., Schiappa R., Laporte B., Rios A., Luxardi G., Kusakabe T., Joly J.S., Darras S., Christiaen L., Contensin M., Auger H., Lamy C., Hudson C., Rothbacher U., Gilchrist M.J., Makabe K.W., Hotta K., Fujiwara S., Satoh N., Satou Y., Lemaire P. (2010). The ANISEED database: digital representation, formalization, and elucidation of a chordate developmental program. Genome Res..

[bib35] Wada H., Kobayashi M., Zhang S.C. (2005). Ets identified as a trans-regulatory factor of amphioxus *Hox*2 by transgenic analysis using ascidian embryos. Dev. Biol..

[bib36] Woolfe A., Goodson M., Goode D.K., Snell P., McEwen G.K., Vavouri T., Smith S.F., North P., Callaway H., Kelly K., Walter K., Abnizova I., Gilks W., Edwards Y.J., Cooke J.E., Elgar G. (2005). Highly conserved non-coding sequences are associated with vertebrate development. PLoS Biol..

