# Supplementary material for: Dissection of a Ciona regulatory element reveals complexity of cross-species enhancer activity
Source: Dev Biol. 2014 Jun 15;390(2):261–72. doi: 10.1016/j.ydbio.2014.03.013 (PMC4010673; doi:10.1016/j.ydbio.2014.03.013)
Supplement: Supplementary file 2 — Supplementary data [file mmc2.doc]

**Supplementary Tables 1-3**

**Supplementary Table 1**

Summary of regulatory interactions identified in at least two vertebrate species.

| **Interaction** | **Mus musculus** | **Gallus gallus** | **Xenopus laevis** | **Danio rerio** |
| --- | --- | --- | --- | --- |
| Pitx3 –(A)-> Foxe3 | Y |  |  | Y |
| Pax6 –(A)-> Six3 | Y |  | Y | Y |
| Foxe3 –(A)-> Pdgfra | Y |  |  | Y |
| Pax6 –(A)-> Cryaa | Y | Y |  |  |
| Creb1 –(A)-> Cryaa | Y | Y |  |  |
| Pax6 –(A)-> Prox1 | Y | Y |  |  |
| Prox1 –(A)-> Crybb1 |  | Y | Y |  |
| Maf –(A)-> Crybb1 | Y | Y |  |  |
| Pax6 –(R)-> Crybb1 |  | Y | Y |  |
| Pax6 –(A)-> Sox2 | Y | Y |  |  |
| Meis1 –(A)-> Pax6 | Y | Y |  |  |
| Six3 –(A)-> Pax6 | Y | Y |  |  |
| Bmp4 –(A)-> Tbx2 | Y |  | Y |  |
| Bmp4 –(A)-> Tbx5 | Y |  | Y |  |
| Pax6 –(A)-> Foxe3 | Y |  | Y |  |
| Pax6 –(A)-> Pax6 | Y |  | Y |  |

**Supplementary Table 2**

Summary of orthologue transposition from the mouse GRN to *Ciona***.**

| **Original Mouse Gene** | **Orthologue (Ciona)** |
| --- | --- |
| Pax6 | Pax6 |
| Sox2 | soxB1 |
| Bmp4 | bmp2/4 |
| Bmp7 | bmp5/7-like |
| Foxe3 | foxE |
| Six3 | six36 |
| Prox1 | prox-a |
| Mab21l1 | NO |
| Msx2 | msxb |
| Sfrp2 | sfrp2 |
| Cryaa | NO |
| Cryab | NO |
| Crygf | Ci-crybg |
| Maf | LOC778757 |
| Rarb | rar |
| Rxrb | rxr |
| Mafb | LOC778757 |
| Mafa | LOC778757 |
| Nrl | LOC778757 |
| Cdkn1b | NO |
| Cdkn1c | NO |
| Pdgfra | NO |
| Dnase2b | NO |
| Snip1 | NO |
| Smad9 | smad1/5 |
| Meis1 | meis |
| Mrg1 | meis |
| Fgfr1 | fgfr |
| Dach1 | NO |
| Sox1 | soxB1 |
| Rax | prx1 |
| Creb1 | creb/atf-c |
| Cryba1 | Ci-crybg |
| Cryba2 | Ci-crybg |
| Cryba4 | Ci-crybg |
| Crybb1 | Ci-crybg |
| Crybb2 | Ci-crybg |
| Crybb3 | Ci-crybg |
| Cryga | Ci-crybg |
| Crygb | Ci-crybg |
| Crygc | Ci-crybg |
| Crygd | Ci-crybg |
| Cryge | Ci-crybg |
| Pou2f1 | pou2 |
| Rybp | NO |
| Itgb1 | LOC100181753 |
|  | LOC100180214 |
|  | LOC100182056 |
| Tcfap2a | ap-2-like2 |
| Itga6 | NO |
| Tbx5 | NO |
| Bfsp1 | NO |
| Ctnnb1 | NO |
| Ccnd1 | NO |
| Cdh1 | NO |
| Crygs | NO |
| Vcan | NO |
| Fgfr3 | NO |
| Aes | NO |
| Hey1 | NO |
| Id2 | NO |
| Id3 | NO |
| Pax2 | NO |
| Pitx3 | NO |
| Rbpj | NO |
| Sox11 | NO |
| Tbx2 | NO |
| Tgfb2 | NO |
| Tle4 | NO |
| Mab21l2 | NO |
| Hsf4 | NO |
| Spag5 | NO |
| Pygo2 | NO |
| Bfsp2 | NO |
| Olfm3 | NO |

**Supplementary Table 3**

Summary of inferred regulatory interactions in *Ciona*, as predicted from the transposed GRN.

| **Original Mouse Gene(s)** | **Ciona Orthologue** | **Upstream Regulator(s)** |
| --- | --- | --- |
| Pax6 | [Pax6](http://www.ncbi.nlm.nih.gov/sites/entrez?Db=gene&Cmd=ShowDetailView&TermToSearch=445643&ordinalpos=2&itool=EntrezSystem2.PEntrez.Gene.Gene_ResultsPanel.Gene_RVDocSum) | [Pax6](http://www.ncbi.nlm.nih.gov/sites/entrez?Db=gene&Cmd=ShowDetailView&TermToSearch=445643&ordinalpos=2&itool=EntrezSystem2.PEntrez.Gene.Gene_ResultsPanel.Gene_RVDocSum), [six36](http://www.ncbi.nlm.nih.gov/sites/entrez?Db=gene&Cmd=ShowDetailView&TermToSearch=445643&ordinalpos=2&itool=EntrezSystem2.PEntrez.Gene.Gene_ResultsPanel.Gene_RVDocSum), [bmp5/7-like](http://www.ncbi.nlm.nih.gov/sites/entrez?Db=gene&Cmd=ShowDetailView&TermToSearch=445643&ordinalpos=2&itool=EntrezSystem2.PEntrez.Gene.Gene_ResultsPanel.Gene_RVDocSum), [meis](http://www.ncbi.nlm.nih.gov/sites/entrez?Db=gene&Cmd=ShowDetailView&TermToSearch=445643&ordinalpos=2&itool=EntrezSystem2.PEntrez.Gene.Gene_ResultsPanel.Gene_RVDocSum), [fgfr](http://www.ncbi.nlm.nih.gov/sites/entrez?Db=gene&Cmd=ShowDetailView&TermToSearch=445643&ordinalpos=2&itool=EntrezSystem2.PEntrez.Gene.Gene_ResultsPanel.Gene_RVDocSum), [soxB1](http://www.ncbi.nlm.nih.gov/sites/entrez?Db=gene&Cmd=ShowDetailView&TermToSearch=445643&ordinalpos=2&itool=EntrezSystem2.PEntrez.Gene.Gene_ResultsPanel.Gene_RVDocSum), [pou2](http://www.ncbi.nlm.nih.gov/sites/entrez?Db=gene&Cmd=ShowDetailView&TermToSearch=445643&ordinalpos=2&itool=EntrezSystem2.PEntrez.Gene.Gene_ResultsPanel.Gene_RVDocSum), [LOC100181753](http://www.ncbi.nlm.nih.gov/sites/entrez?Db=gene&Cmd=ShowDetailView&TermToSearch=445643&ordinalpos=2&itool=EntrezSystem2.PEntrez.Gene.Gene_ResultsPanel.Gene_RVDocSum), [LOC100180214](http://www.ncbi.nlm.nih.gov/sites/entrez?Db=gene&Cmd=ShowDetailView&TermToSearch=445643&ordinalpos=2&itool=EntrezSystem2.PEntrez.Gene.Gene_ResultsPanel.Gene_RVDocSum), [LOC100182056](http://www.ncbi.nlm.nih.gov/sites/entrez?Db=gene&Cmd=ShowDetailView&TermToSearch=445643&ordinalpos=2&itool=EntrezSystem2.PEntrez.Gene.Gene_ResultsPanel.Gene_RVDocSum), [prx1](http://www.ncbi.nlm.nih.gov/sites/entrez?Db=gene&Cmd=ShowDetailView&TermToSearch=445643&ordinalpos=2&itool=EntrezSystem2.PEntrez.Gene.Gene_ResultsPanel.Gene_RVDocSum) |
| Six3 | [six36](http://www.ncbi.nlm.nih.gov/sites/entrez?Db=gene&Cmd=ShowDetailView&TermToSearch=778753&ordinalpos=2&itool=EntrezSystem2.PEntrez.Gene.Gene_ResultsPanel.Gene_RVDocSum) | [Pax6](http://www.ncbi.nlm.nih.gov/sites/entrez?Db=gene&Cmd=ShowDetailView&TermToSearch=778753&ordinalpos=2&itool=EntrezSystem2.PEntrez.Gene.Gene_ResultsPanel.Gene_RVDocSum), [fgfr](http://www.ncbi.nlm.nih.gov/sites/entrez?Db=gene&Cmd=ShowDetailView&TermToSearch=778753&ordinalpos=2&itool=EntrezSystem2.PEntrez.Gene.Gene_ResultsPanel.Gene_RVDocSum) |
| Prox1 | [prox-a](http://www.ncbi.nlm.nih.gov/sites/entrez?Db=gene&Cmd=ShowDetailView&TermToSearch=778983&ordinalpos=2&itool=EntrezSystem2.PEntrez.Gene.Gene_ResultsPanel.Gene_RVDocSum) | [Pax6](http://www.ncbi.nlm.nih.gov/sites/entrez?Db=gene&Cmd=ShowDetailView&TermToSearch=778983&ordinalpos=2&itool=EntrezSystem2.PEntrez.Gene.Gene_ResultsPanel.Gene_RVDocSum), [foxE](http://www.ncbi.nlm.nih.gov/sites/entrez?Db=gene&Cmd=ShowDetailView&TermToSearch=778983&ordinalpos=2&itool=EntrezSystem2.PEntrez.Gene.Gene_ResultsPanel.Gene_RVDocSum), [LOC100181753](http://www.ncbi.nlm.nih.gov/sites/entrez?Db=gene&Cmd=ShowDetailView&TermToSearch=778983&ordinalpos=2&itool=EntrezSystem2.PEntrez.Gene.Gene_ResultsPanel.Gene_RVDocSum), [LOC100180214](http://www.ncbi.nlm.nih.gov/sites/entrez?Db=gene&Cmd=ShowDetailView&TermToSearch=778983&ordinalpos=2&itool=EntrezSystem2.PEntrez.Gene.Gene_ResultsPanel.Gene_RVDocSum), [LOC100182056](http://www.ncbi.nlm.nih.gov/sites/entrez?Db=gene&Cmd=ShowDetailView&TermToSearch=778983&ordinalpos=2&itool=EntrezSystem2.PEntrez.Gene.Gene_ResultsPanel.Gene_RVDocSum) |
| Foxe3 | [foxE](http://www.ncbi.nlm.nih.gov/sites/entrez?Db=gene&Cmd=ShowDetailView&TermToSearch=445746&ordinalpos=2&itool=EntrezSystem2.PEntrez.Gene.Gene_ResultsPanel.Gene_RVDocSum) | [smad1/5](http://www.ncbi.nlm.nih.gov/sites/entrez?Db=gene&Cmd=ShowDetailView&TermToSearch=445746&ordinalpos=2&itool=EntrezSystem2.PEntrez.Gene.Gene_ResultsPanel.Gene_RVDocSum), [prx1](http://www.ncbi.nlm.nih.gov/sites/entrez?Db=gene&Cmd=ShowDetailView&TermToSearch=445746&ordinalpos=2&itool=EntrezSystem2.PEntrez.Gene.Gene_ResultsPanel.Gene_RVDocSum), [Pax6](http://www.ncbi.nlm.nih.gov/sites/entrez?Db=gene&Cmd=ShowDetailView&TermToSearch=445746&ordinalpos=2&itool=EntrezSystem2.PEntrez.Gene.Gene_ResultsPanel.Gene_RVDocSum) |
| Bmp4 | [bmp2/4](http://www.ncbi.nlm.nih.gov/sites/entrez?Db=gene&Cmd=ShowDetailView&TermToSearch=778554&ordinalpos=2&itool=EntrezSystem2.PEntrez.Gene.Gene_ResultsPanel.Gene_RVDocSum) | [fgfr](http://www.ncbi.nlm.nih.gov/sites/entrez?Db=gene&Cmd=ShowDetailView&TermToSearch=778554&ordinalpos=2&itool=EntrezSystem2.PEntrez.Gene.Gene_ResultsPanel.Gene_RVDocSum) |
| Sox2, Sox1 | [soxB1](http://www.ncbi.nlm.nih.gov/sites/entrez?Db=gene&Cmd=ShowDetailView&TermToSearch=445713&ordinalpos=2&itool=EntrezSystem2.PEntrez.Gene.Gene_ResultsPanel.Gene_RVDocSum) | [bmp2/4](http://www.ncbi.nlm.nih.gov/sites/entrez?Db=gene&Cmd=ShowDetailView&TermToSearch=445713&ordinalpos=2&itool=EntrezSystem2.PEntrez.Gene.Gene_ResultsPanel.Gene_RVDocSum), [Pax6](http://www.ncbi.nlm.nih.gov/sites/entrez?Db=gene&Cmd=ShowDetailView&TermToSearch=445713&ordinalpos=2&itool=EntrezSystem2.PEntrez.Gene.Gene_ResultsPanel.Gene_RVDocSum), [bmp5/7-like](http://www.ncbi.nlm.nih.gov/sites/entrez?Db=gene&Cmd=ShowDetailView&TermToSearch=445713&ordinalpos=2&itool=EntrezSystem2.PEntrez.Gene.Gene_ResultsPanel.Gene_RVDocSum) |
| Msx2 | [msxb](http://www.ncbi.nlm.nih.gov/sites/entrez?Db=gene&Cmd=ShowDetailView&TermToSearch=445677&ordinalpos=2&itool=EntrezSystem2.PEntrez.Gene.Gene_ResultsPanel.Gene_RVDocSum) | [bmp2/4](http://www.ncbi.nlm.nih.gov/sites/entrez?Db=gene&Cmd=ShowDetailView&TermToSearch=445677&ordinalpos=2&itool=EntrezSystem2.PEntrez.Gene.Gene_ResultsPanel.Gene_RVDocSum) |
| Bmp7 | [bmp5/7-like](http://www.ncbi.nlm.nih.gov/sites/entrez?Db=gene&Cmd=ShowDetailView&TermToSearch=778555&ordinalpos=2&itool=EntrezSystem2.PEntrez.Gene.Gene_ResultsPanel.Gene_RVDocSum) |  |
| Sfrp2 | [sfrp2](http://www.ncbi.nlm.nih.gov/sites/entrez?Db=gene&Cmd=ShowDetailView&TermToSearch=778984&ordinalpos=2&itool=EntrezSystem2.PEntrez.Gene.Gene_ResultsPanel.Gene_RVDocSum) | [Pax6](http://www.ncbi.nlm.nih.gov/sites/entrez?Db=gene&Cmd=ShowDetailView&TermToSearch=778984&ordinalpos=2&itool=EntrezSystem2.PEntrez.Gene.Gene_ResultsPanel.Gene_RVDocSum), [bmp5/7-like](http://www.ncbi.nlm.nih.gov/sites/entrez?Db=gene&Cmd=ShowDetailView&TermToSearch=778984&ordinalpos=2&itool=EntrezSystem2.PEntrez.Gene.Gene_ResultsPanel.Gene_RVDocSum) |
| Smad9 | [smad1/5](http://www.ncbi.nlm.nih.gov/sites/entrez?Db=gene&Cmd=ShowDetailView&TermToSearch=778754&ordinalpos=2&itool=EntrezSystem2.PEntrez.Gene.Gene_ResultsPanel.Gene_RVDocSum) |  |
| Meis1, Mrg1 | [meis](http://www.ncbi.nlm.nih.gov/sites/entrez?Db=gene&Cmd=ShowDetailView&TermToSearch=778904&ordinalpos=2&itool=EntrezSystem2.PEntrez.Gene.Gene_ResultsPanel.Gene_RVDocSum) |  |
| Fgfr1 | [fgfr](http://www.ncbi.nlm.nih.gov/sites/entrez?Db=gene&Cmd=ShowDetailView&TermToSearch=445706&ordinalpos=2&itool=EntrezSystem2.PEntrez.Gene.Gene_ResultsPanel.Gene_RVDocSum) |  |
| Maf, Mafa, Mafb, Nrl | [LOC778757](http://www.ncbi.nlm.nih.gov/sites/entrez?Db=gene&Cmd=ShowDetailView&TermToSearch=778757&ordinalpos=2&itool=EntrezSystem2.PEntrez.Gene.Gene_ResultsPanel.Gene_RVDocSum) | [LOC778757](http://www.ncbi.nlm.nih.gov/sites/entrez?Db=gene&Cmd=ShowDetailView&TermToSearch=778757&ordinalpos=2&itool=EntrezSystem2.PEntrez.Gene.Gene_ResultsPanel.Gene_RVDocSum), [Pax6](http://www.ncbi.nlm.nih.gov/sites/entrez?Db=gene&Cmd=ShowDetailView&TermToSearch=778757&ordinalpos=2&itool=EntrezSystem2.PEntrez.Gene.Gene_ResultsPanel.Gene_RVDocSum), [LOC100181753](http://www.ncbi.nlm.nih.gov/sites/entrez?Db=gene&Cmd=ShowDetailView&TermToSearch=778757&ordinalpos=2&itool=EntrezSystem2.PEntrez.Gene.Gene_ResultsPanel.Gene_RVDocSum), [LOC100180214](http://www.ncbi.nlm.nih.gov/sites/entrez?Db=gene&Cmd=ShowDetailView&TermToSearch=778757&ordinalpos=2&itool=EntrezSystem2.PEntrez.Gene.Gene_ResultsPanel.Gene_RVDocSum), [LOC100182056](http://www.ncbi.nlm.nih.gov/sites/entrez?Db=gene&Cmd=ShowDetailView&TermToSearch=778757&ordinalpos=2&itool=EntrezSystem2.PEntrez.Gene.Gene_ResultsPanel.Gene_RVDocSum) |
| Crygf, Cryba1, Cryba2, Cryba4, Crybb1, Crybb2, Crybb3, Cryga, Crygb, Crygc, Crygd, Cryge | [Ci-crybg](http://www.ncbi.nlm.nih.gov/sites/entrez?Db=gene&Cmd=ShowDetailView&TermToSearch=100175621&ordinalpos=2&itool=EntrezSystem2.PEntrez.Gene.Gene_ResultsPanel.Gene_RVDocSum) | [LOC778757](http://www.ncbi.nlm.nih.gov/sites/entrez?Db=gene&Cmd=ShowDetailView&TermToSearch=100175621&ordinalpos=2&itool=EntrezSystem2.PEntrez.Gene.Gene_ResultsPanel.Gene_RVDocSum), [soxB1](http://www.ncbi.nlm.nih.gov/sites/entrez?Db=gene&Cmd=ShowDetailView&TermToSearch=100175621&ordinalpos=2&itool=EntrezSystem2.PEntrez.Gene.Gene_ResultsPanel.Gene_RVDocSum), [rar](http://www.ncbi.nlm.nih.gov/sites/entrez?Db=gene&Cmd=ShowDetailView&TermToSearch=100175621&ordinalpos=2&itool=EntrezSystem2.PEntrez.Gene.Gene_ResultsPanel.Gene_RVDocSum), [rxr](http://www.ncbi.nlm.nih.gov/sites/entrez?Db=gene&Cmd=ShowDetailView&TermToSearch=100175621&ordinalpos=2&itool=EntrezSystem2.PEntrez.Gene.Gene_ResultsPanel.Gene_RVDocSum), [six36](http://www.ncbi.nlm.nih.gov/sites/entrez?Db=gene&Cmd=ShowDetailView&TermToSearch=100175621&ordinalpos=2&itool=EntrezSystem2.PEntrez.Gene.Gene_ResultsPanel.Gene_RVDocSum), [Pax6](http://www.ncbi.nlm.nih.gov/sites/entrez?Db=gene&Cmd=ShowDetailView&TermToSearch=100175621&ordinalpos=2&itool=EntrezSystem2.PEntrez.Gene.Gene_ResultsPanel.Gene_RVDocSum), [prox-a](http://www.ncbi.nlm.nih.gov/sites/entrez?Db=gene&Cmd=ShowDetailView&TermToSearch=100175621&ordinalpos=2&itool=EntrezSystem2.PEntrez.Gene.Gene_ResultsPanel.Gene_RVDocSum) |
| Rarb | [rar](http://www.ncbi.nlm.nih.gov/sites/entrez?Db=gene&Cmd=ShowDetailView&TermToSearch=780719&ordinalpos=2&itool=EntrezSystem2.PEntrez.Gene.Gene_ResultsPanel.Gene_RVDocSum) |  |
| Rxrb | [rxr](http://www.ncbi.nlm.nih.gov/sites/entrez?Db=gene&Cmd=ShowDetailView&TermToSearch=778746&ordinalpos=2&itool=EntrezSystem2.PEntrez.Gene.Gene_ResultsPanel.Gene_RVDocSum) |  |
| Rax | [prx1](http://www.ncbi.nlm.nih.gov/sites/entrez?Db=gene&Cmd=ShowDetailView&TermToSearch=445693&ordinalpos=2&itool=EntrezSystem2.PEntrez.Gene.Gene_ResultsPanel.Gene_RVDocSum) |  |
| Pou2f1 | [pou2](http://www.ncbi.nlm.nih.gov/sites/entrez?Db=gene&Cmd=ShowDetailView&TermToSearch=778732&ordinalpos=2&itool=EntrezSystem2.PEntrez.Gene.Gene_ResultsPanel.Gene_RVDocSum) |  |
| Itgb1 | [LOC100181753](http://www.ncbi.nlm.nih.gov/sites/entrez?Db=gene&Cmd=ShowDetailView&TermToSearch=100181753&ordinalpos=2&itool=EntrezSystem2.PEntrez.Gene.Gene_ResultsPanel.Gene_RVDocSum) |  |
|  | [LOC100180214](http://www.ncbi.nlm.nih.gov/sites/entrez?Db=gene&Cmd=ShowDetailView&TermToSearch=100180214&ordinalpos=2&itool=EntrezSystem2.PEntrez.Gene.Gene_ResultsPanel.Gene_RVDocSum) |  |
|  | [LOC100182056](http://www.ncbi.nlm.nih.gov/sites/entrez?Db=gene&Cmd=ShowDetailView&TermToSearch=100182056&ordinalpos=2&itool=EntrezSystem2.PEntrez.Gene.Gene_ResultsPanel.Gene_RVDocSum) |  |
